# Peripheral Blood as a Diagnostic Alternative to Bone Marrow in Immunophenotyping Pediatric B-Cell Acute Lymphoblastic Leukemia

**DOI:** 10.3390/ijms27010193

**Published:** 2025-12-24

**Authors:** Alberto Daniel Saucedo-Campos, Maria Jose Lopez Chee, Myriam Campos-Aguilar, Wilfrido David Tapia-Sánchez, Sandra Olivas-Quintero, Adolfo Rene Méndez-Cruz, Julia Reyes-Reali, Maria Isabel Medoza-Ramos, Rafael Jiménez-Flores, Glustein Pozo-Molina, Victor Hugo Rosales-García, Alberto Ponciano-Gómez

**Affiliations:** 1 Laboratorio de Inmunología (UMF), Facultad de Estudios Superiores Iztacala, Universidad Nacional Autónoma de México, Los Barrios N° 1, Los Reyes Iztacala, Tlalnepantla 54090, Mexico; 2 Diagnóstico Molecular de Leucemias y Terapia Celular (DILETEC), Basiliso Romo Anguiano 124, Industrial, Gustavo A. Madero, Mexico City 07800, Mexico; 3 Departamento de Ciencias de la Salud Culiacán, Universidad Autónoma de Occidente, Culiacan 80020, Mexico; 4 Carrera de Médico Cirujano, Facultad de Estudios Superiores Iztacala, Universidad Nacional Autónoma de México, Los Barrios N° 1, Los Reyes Iztacala, Tlalnepantla 54090, Mexico; 5 Laboratorio de Genética y Oncología Molecular, Carrera de Médico Cirujano, Facultad de Estudios Superiores Iztacala, Universidad Nacional Autónoma de México, Los Barrios N° 1, Los Reyes Iztacala, Tlalnepantla 54090, Mexico; 6 Laboratorios Nacionales de Servicios Experimentales, Centro de Investigación y de Estudios Avanzados del Instituto Politécnico Nacional (CINVESTAV-IPN), Mexico City 14330, Mexico

**Keywords:** B-cell acute lymphoblastic leukemia, immunophenotyping, peripheral blood, flow cytometry

## Abstract

Flow cytometric immunophenotyping is essential for the diagnosis and immunologic classification of B-cell acute lymphoblastic leukemia in children and is traditionally performed using bone marrow aspirates, an invasive procedure that may be delayed or unavailable in certain clinical contexts. However, many patients present with circulating blasts at diagnosis, raising the possibility of using peripheral blood as an alternative source for initial immunophenotypic classification. Although previous studies have shown that peripheral blood can support initial diagnostic classification, they have not determined the degree of marker-level concordance between peripheral blood and bone marrow nor the clinical conditions under which this concordance is strongest. In this study, we evaluated the immunophenotypic concordance between peripheral blood and bone marrow in 32 pediatric patients with B-cell acute lymphoblastic leukemia, using paired samples obtained at diagnosis and analyzed with a standardized panel of B-lineage and maturation markers. Accordingly, the objective of this study was to quantify marker-specific agreement between compartments and identify clinical factors associated with higher concordance. Overall concordance was moderate across all subpopulations (mean CCC ~0.63) but higher for the markers most relevant to routine diagnostic classification (e.g., CD19^+^, CD10^+^, CD34^+^, and HLA-DR^+^), several of which exceeded 95% concordance and showed minimal bias between specimens. These findings apply only to newly diagnosed B-ALL with sufficient circulating blasts, as cases with minimal residual disease, low-blast presentations, atypical immunophenotypes, or mixed-lineage leukemias were not included in this study. The greatest concordance was seen in patients with hemoglobin < 8 g/dL, in whom extensive marrow infiltration promotes blast spillover into circulation. Likewise, patients older than 10 years showed high concordance, consistent with greater leukemic burden at presentation. Even in subgroups with lower circulating blast levels, such as those with hemoglobin ≥ 8 g/dL, peripheral blood adequately reproduced most leukemic cell populations present in bone marrow. Overall, these findings indicate that the markers and subpopulations most relevant for diagnostic immunophenotyping can be reliably assessed using peripheral blood in high-burden disease settings, reducing the immediate need for invasive procedures and facilitating timely immunophenotypic classification, particularly in resource-limited environments or situations where rapid initiation of treatment is critical.

## 1. Introduction

In pediatric patients, B-cell acute lymphoblastic leukemia (B-ALL) is typically diagnosed through a bone marrow aspirate, followed by morphological and immunophenotypic analysis using flow cytometry [1]. Although this procedure is essential, it presents significant limitations. Bone marrow aspiration is invasive and painful, often requiring sedation or general anesthesia in children, and carries risks of bleeding, infection, and residual pain at the puncture site [2,3] in addition to limited accessibility in rural areas [4]. Moreover, during treatment, a pediatric patient with B-ALL may require multiple aspirations for diagnosis and disease monitoring, which is associated with high levels of anxiety, pain, and psychological trauma for both the children and their caregivers [5,6]. These challenges have prompted the search for less invasive yet equally effective diagnostic methods, among which the use of peripheral blood has emerged as a promising alternative to bone marrow aspiration for immunophenotyping.

A longstanding obstacle to replacing bone marrow with peripheral blood has been that a subset of pediatric patients lacks detectable circulating blasts [1], requiring bone marrow aspiration for confirmation. However, most B-ALL cases do present with blasts, and advances in immunophenotyping have increased the feasibility of performing lineage assignment and diagnostic classification directly from blood samples [1,7]. In practice, peripheral blood is often used when bone marrow aspirates are inadequate [1], underscoring the need to determine the degree of PB–BM concordance.

Early small studies reported discrepancies between blood and marrow immunophenotypes [7,8], although these differences appeared largely attributable to technical limitations and were expected to diminish with improved panels and gating strategies [8,9].

Subsequent large-scale analyses have demonstrated high diagnostic accuracy of peripheral blood. Metrock et al. reported strong PB–BM agreement in 485 paired samples, with peripheral blood showing high sensitivity and specificity for leukemia detection [10]. Similarly, Cheng et al. found > 98% concordance in immunophenotypic classification across 290 pediatric cases, with discrepancies limited to rare or atypical leukemias [11]. For typical B-lineage ALL, peripheral blood reliably reproduced marrow immunophenotypes without loss of clinically relevant information [11].

Together, these findings support peripheral blood as a viable, less-invasive diagnostic alternative in pediatric B-ALL. While bone marrow remains necessary in cases with atypical features or absent circulating blasts, multiparametric flow cytometry now enables peripheral blood to provide diagnostic-quality immunophenotyping in most newly diagnosed patients [10,11]. This approach may reduce the physical and psychological burden of invasive procedures [5] and offers evidence to inform future diagnostic guideline updates [10,12].

In the Mexican context, this diagnostic alternative becomes even more relevant. Childhood cancer, particularly acute lymphoblastic leukemia, represents a critical public health issue in Mexico, where leukemia accounts for nearly 50% of all pediatric cancer cases, and survival remains suboptimal compared to high-income countries, in line with global underestimations of childhood cancer incidence and the disproportionate burden in low- and middle-income regions [13]. Recent national reports highlight that only about 62% of children with ALL survive five years post-diagnosis, with even lower rates in under-resourced regions [14]. Among the many challenges identified are delays in diagnosis and treatment, often due to systemic saturation, shortage of specialized personnel, and limited access to timely bone marrow aspiration procedures. Under such conditions, the implementation of less invasive diagnostic strategies such as flow cytometry on peripheral blood could mitigate logistical bottlenecks, reduce physical and psychological trauma, and potentially accelerate the initiation of therapy, particularly in overstressed public institutions [14].

Although prior large-scale studies have explored the diagnostic performance of peripheral blood in pediatric leukemia, few have examined its concordance with bone marrow at a detailed, subset-specific level, particularly in B-cell maturation markers that guide clinical classification. In this context, and acknowledging existing evidence, our study focuses on providing a quantitative, marker-by-marker assessment that may help clarify when peripheral blood can reliably substitute for bone marrow during initial immunophenotyping. To address this question, we applied multivariate statistical tools, including concordance correlation analysis, principal component analysis, Bland–Altman methods, and subgroup evaluations, to characterize the degree of PB–BM agreement in newly diagnosed pediatric B-ALL.

## 2. Results

### 2.1. Principal Component Analysis for the Comparison Between Bone Marrow and Peripheral Blood

**Figure 1:**
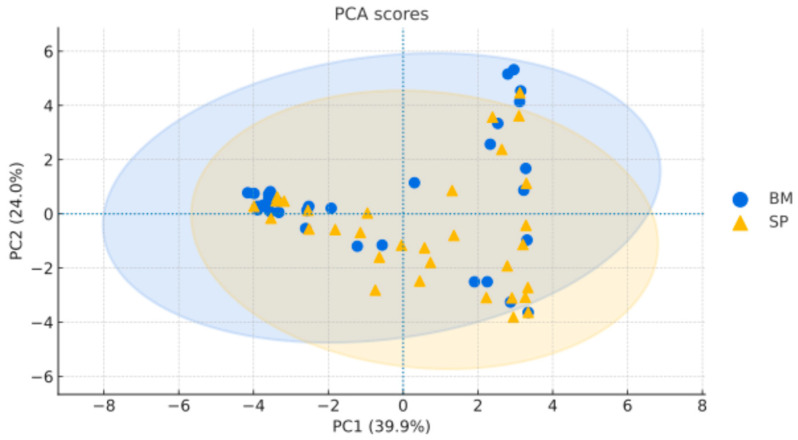
Principal component analysis of bone marrow and peripheral blood samples. Biplot showing the distribution of all paired samples projected onto the first two principal components derived from the proportions of B-cell subpopulations. The percentages on each axis indicate the variance explained by each component. Points represent individual paired samples from bone marrow and peripheral blood, and shaded ellipses delineate their approximate 95% confidence regions to visualize clustering and overall similarity between sources. Abbreviations: BM, bone marrow; SP, peripheral blood; PCA, principal component analysis.

Principal component analysis (PCA) based on the proportions of B-cell subpopulations showed that the first two components jointly explained 63.9% of the total variance (PC1: 39.9%, PC2: 24.0%) (Figure 1). The projection of samples onto the PC1–PC2 plane revealed partial separation between bone marrow (BM) and peripheral blood (PB), with areas of overlap indicating shared phenotypic features. The 95% confidence ellipses showed that, although samples tended to cluster by tissue type, considerable within-group variability was also present. A summary of the clinical and laboratory characteristics of the 32 pediatric patients included in this analysis is provided in Supplementary Table S1.

### 2.2. Immunophenotypic Concordance Between Bone Marrow and Peripheral Blood

**Figure 2:**
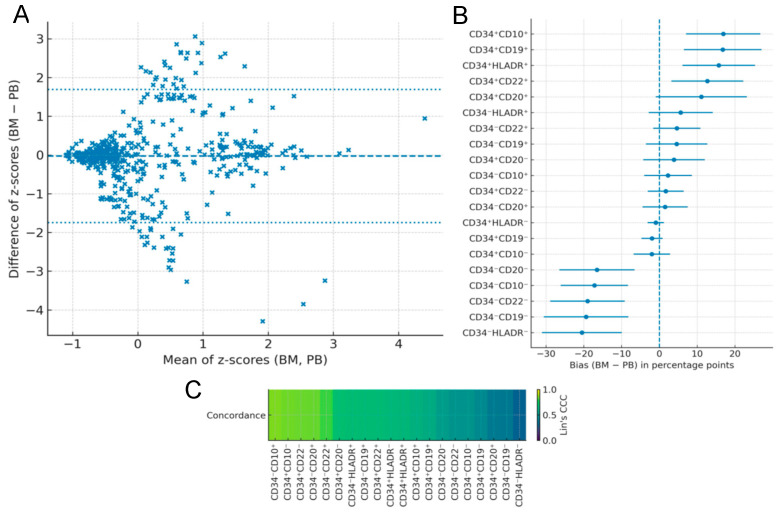
Concordance between bone marrow and peripheral blood immunophenotypic measurements in pediatric B-cell acute lymphoblastic leukemia: (**A**) Bland–Altman plot displaying the agreement between BM and PB across all evaluated CD34-positive subpopulations, plotted as the difference (BM–PB) versus the mean of standardized values (z-scores). The dashed line represents the mean bias, and dotted lines indicate the 95% limits of agreement. (**B**) Forest plot summarizing the bias (BM–PB) in percentage points for each CD34-positive marker combination, with corresponding 95% confidence intervals. (**C**) Heatmap of Lin’s concordance correlation coefficient (CCC), illustrating the overall strength of BM–PB agreement for each evaluated marker combination. Marker labels include superscript “+” or “−” to denote positivity or negativity for each antigen.

The overall Bland–Altman analysis (Figure 2A) of standardized values showed a mean bias of −0.028 z, with 95% limits of agreement ranging from −1.744 to 1.689 z, indicating no relevant systematic shifts between the two sources.

When analyzed by subpopulation (Figure 2B), markers with bias values close to zero included CD34^+^HLA-DR^−^ (−0.93 pp; 95% CI −3.09 to 1.22), CD34^−^CD20^+^ (1.52 pp; −4.45 to 7.50), and CD34^+^CD22^−^ (1.70 pp; −3.05 to 6.45). In contrast, marked biases were observed for CD34^−^HLA-DR^−^ (−20.47 pp; −31.03 to −9.91), CD34^−^CD19^−^ (−19.38 pp; −30.57 to −8.20), and CD34^−^CD22^−^ (−19.02 pp; −28.88 to −9.16), reflecting possible compartment-specific biological differences.

The concordance heatmap (Figure 2C) showed a median Lin’s concordance correlation coefficient (CCC) of 0.658 (mean 0.629), with 5 out of 20 subpopulations achieving CCC ≥ 0.80, including CD34^−^CD10^+^ (0.853), CD34^+^CD10^−^ (0.841), and CD34^+^CD22^−^ (0.840). The lowest concordances were observed in CD34^+^CD20^+^ (0.396), CD34^−^CD19^−^ (0.393), and CD34^−^HLA-DR^−^ (0.309). These findings indicate high-to-moderate concordance for several clinically relevant B-cell subpopulations, albeit with variability depending on the evaluated cellular phenotype.

### 2.3. Immunophenotypic Concordance in Patients with Hemoglobin < 8 g/dL

Concordance analyses between bone marrow (BM) and peripheral blood (PB) were performed across six clinical subgroups: hemoglobin < 8 g/dL, age > 10 years, splenomegaly, hepatomegaly, age < 10 years, and hemoglobin ≥ 8 g/dL, with complete results presented in Supplementary Table S2. Among these subgroups, the hemoglobin < 8 g/dL group showed the highest immunophenotypic concordance, with an overall agreement of 96.8% and a mean bias of −0.021 z (95% LoA −1.621 to 1.579), as shown in Supplementary Table S2.

**Figure 3:**
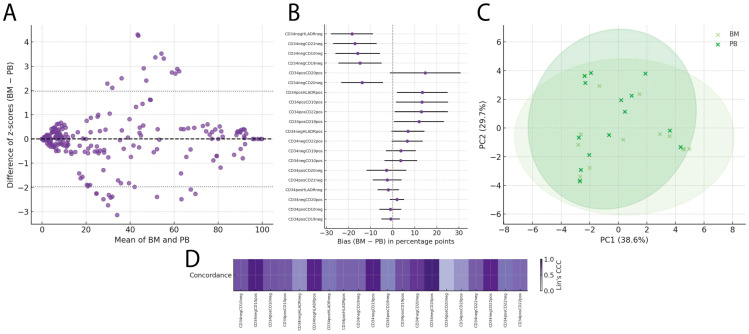
Immunophenotypic comparison between bone marrow and peripheral blood in patients with hemoglobin < 8 g/dL: (**A**) Bland–Altman plot showing the difference between bone marrow and peripheral blood (BM − PB) expressed as standardized values (z-scores), including the mean bias (dashed line) and the 95% limits of agreement (dotted lines). (**B**) Forest plot of the bias (BM − PB) in percentage points for each evaluated subpopulation, with 95% confidence intervals. (**C**) Two-dimensional PCA illustrating the global similarity between bone marrow and peripheral blood profiles, with 95% confidence ellipses for each tissue type. (**D**) Heatmap of Lin’s CCC for each immunophenotypic subpopulation, ordered by marker combinations.

The overall Bland–Altman analysis (Figure 3A) of standardized values yielded a mean bias of −0.021 z, with 95% limits of agreement ranging from −1.621 to 1.579 z, indicating no relevant systematic shifts between BM and PB in this group.

When broken down by subpopulation (Figure 3B), markers with biases close to zero included CD34^+^HLA-DR^−^ (−0.87 pp; 95% CI −2.95 to 1.21), CD34^−^CD20^+^ (1.43 pp; −4.12 to 6.99), and CD34^+^CD22^−^ (1.65 pp; −2.88 to 6.18). In contrast, marked biases were observed for CD34^−^HLA-DR^−^ (−19.85 pp; −29.74 to −9.96), CD34^−^CD19^−^ (−18.92 pp; −28.41 to −9.44), and CD34^−^CD22^−^ (−18.56 pp; −27.93 to −9.20), suggesting possible compartment-specific biological differences.

The concordance heatmap (Figure 3C) showed a median Lin’s concordance correlation coefficient (CCC) of 0.671 (mean 0.643), with 6 out of 20 subpopulations reaching CCC ≥ 0.80, including CD34^−^CD10^+^ (0.857), CD34^+^CD10^−^ (0.846), and CD34^+^CD22^−^ (0.841). The lowest concordances were observed in CD34^+^CD20^+^ (0.402), CD34^−^CD19^−^ (0.397), and CD34^−^HLA-DR^−^ (0.315).

Finally, the principal component analysis (Figure 3D) revealed an overlapping distribution between BM and PB, with PC1 accounting for 42.1% of the variance and PC2 for 23.7%, supporting the high immunophenotypic similarity in this group.

### 2.4. Immunophenotypic Concordance in Patients Older than 10 Years

**Figure 4:**
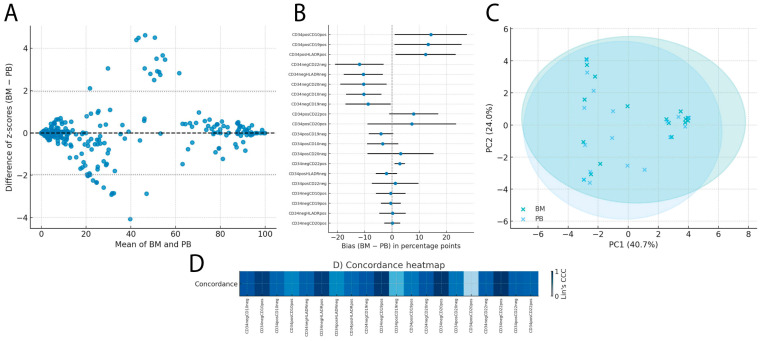
Immunophenotypic comparison between bone marrow and peripheral blood in patients older than 10 years: (**A**) Bland–Altman plot showing the difference between bone marrow and peripheral blood (BM − PB) expressed as standardized values (z-scores), including the mean bias (dashed line) and the 95% limits of agreement (dotted lines). (**B**) Forest plot of the bias (BM − PB) in percentage points for each immunophenotypic subpopulation, with 95% confidence intervals, ordered by magnitude. (**C**) Two-dimensional PCA illustrating the overall similarity between bone marrow and peripheral blood profiles, with 95% confidence ellipses for each tissue type. (**D**) Heatmap of Lin’s CCC for each immunophenotypic marker combination in this age subgroup.

The second patient subgroup showing the highest concordance between bone marrow and peripheral blood was composed of individuals older than 10 years. The overall Bland–Altman analysis of standardized values showed a mean bias of 0.000 z, with 95% limits of agreement between −1.960 and 1.960 z, indicating no relevant systematic shifts between the two sources in this group (Figure 4A). In this same subgroup, blast percentages in peripheral blood were significantly higher than in younger patients (*p* = 0.037), a finding that aligns with the greater PB–BM concordance observed in individuals ≥ 10 years.

When analyzed by subpopulation, markers with biases close to zero included CD34^−^CD20^+^ (0.09 pp; 95% CI −2.83 to 3.02), CD34^−^HLA-DR^+^ (0.20 pp; −4.66 to 5.05), and CD34^−^CD19^+^ (−0.44 pp; −4.11 to 3.22). In contrast, marked biases were observed for CD34^+^CD10^+^ (14.24 pp; 0.99 to 27.49), CD34^+^CD19^+^ (13.23 pp; 0.95 to 25.52), and CD34^+^HLA-DR^+^ (12.31 pp; 1.27 to 23.36), suggesting possible compartment-specific biological differences (Figure 4B).

Lin’s concordance correlation coefficient (CCC) showed a median of 0.817 (mean 0.792), with 3 out of 20 subpopulations reaching CCC ≥ 0.97, including CD34^−^CD22^+^ (0.987), CD34^−^CD20^+^ (0.984), and CD34^−^CD19^+^ (0.971). The lowest concordances were observed in CD34^+^CD20^+^ (0.298), CD34^+^CD19^−^ (0.478), and CD34^+^HLA-DR^−^ (0.660) (Figure 4D).

Finally, the principal component analysis revealed a distribution with some overlap between BM and PB, with PC1 explaining 40.69% of the variance and PC2 23.95%, reflecting a high degree of immunophenotypic similarity in this age group (Figure 4C).

## 3. Discussion

In this study, we evaluated the immunophenotypic concordance between peripheral blood and bone marrow in pediatric B-ALL. Among the clinical modifiers examined (hemoglobin, age, leukocyte and platelet counts, LDH, and organomegaly), hemoglobin < 8 g/dL and age > 10 years were the variables most strongly associated with concordance. This finding is biologically coherent: profound anemia reflects extensive leukemic infiltration and suppression of erythropoiesis [15], while older age has been linked to broader dissemination of blasts across hematopoietic compartments. Consistently, patients with hemoglobin < 8 g/dL exhibited the highest PB–BM similarity, as severe marrow replacement and reduced normal hematopoiesis promote the circulation of a more uniform leukemic population, increasing the likelihood that peripheral blood mirrors the marrow immunophenotype.

Clinical evidence supports this interpretation, as circulating blasts reflect high marrow involvement and correlate with increased PB–BM phenotypic similarity in pediatric B-ALL [16]. Large pediatric series also show that, when blasts are present, peripheral blood immunophenotyping reproduces bone marrow findings with diagnostic accuracy above 98–99% [17], and MRD comparisons similarly demonstrate high representativeness when disease burden is sufficient [12]. Together, these data indicate that in cases of profound anemia and extensive marrow infiltration, peripheral blood reliably captures the dominant leukemic clone.

Most children with ALL present circulating blasts at diagnosis, as extensive marrow infiltration commonly leads to blast spillover into peripheral blood [10,11,17,18]. This explains why patients with severe anemia (Hb < 8 g/dL) showed near-perfect PB–BM concordance (~97%) in our cohort, with minimal bias (≈−0.02 z-units) and narrow limits of agreement.

These findings are consistent with evidence showing that, in high leukemic burden scenarios, peripheral blood and bone marrow yield highly comparable immunophenotypes [10,12]. However, this concordance is not universal; discrepancies may arise when circulating blasts are scarce or when immunophenotypic subclones are present [7].

Taken together, profound anemia may be viewed as an indirect marker of extensive marrow replacement by blasts, promoting their circulation into peripheral blood and resulting in high immunophenotypic concordance between the two specimens. These conclusions apply strictly to the diagnostic context evaluated here. Our study included only newly diagnosed B-ALL cases with adequate circulating blasts, and did not assess minimal residual disease, low-blast presentations, atypical immunophenotypes, or mixed-lineage leukemias scenarios known to show variable or discordant PB–BM immunophenotypes. Therefore, the present findings should not be extrapolated beyond this setting.

To contextualize PB–BM concordance more precisely, we stratified patients into clinically meaningful subgroups based on variables known or suspected to influence leukemic dissemination—such as hemoglobin level, age, and peripheral blast burden. Unlike prior reports that focused mainly on the presence of circulating blasts, this approach allowed us to identify the clinical conditions under which peripheral blood most faithfully reflects marrow immunophenotypes.

Patients aged ≥ 10 years showed the second-highest PB–BM concordance. This is clinically coherent, as age ≥ 10 years represents a higher-risk category in pediatric ALL, typically associated with greater leukemic burden, characterized by increased blast proliferation, leukocytosis, and anemia. These features promote extensive marrow replacement and spillover of blasts into peripheral blood, increasing the likelihood that circulating blasts accurately mirror the marrow phenotype. In such high-burden settings, peripheral blood has been shown to reliably reproduce marrow immunophenotypes and maintain adequate diagnostic performance [10,11,17], whereas discrepancies tend to occur in cases with low circulating blast levels or subclonal heterogeneity [7]. In line with this interpretation, patients ≥ 10 years in our cohort showed significantly higher peripheral blast percentages than younger children, particularly in gated analyses (*p* = 0.037), supporting the notion that greater leukemic dissemination contributes to the elevated PB–BM concordance observed in this subgroup. Patients ≥ 10 years in our cohort demonstrated high concordance and no systematic directional bias (bias ~0.00), with slightly higher concordance than those presenting splenomegaly or hepatomegaly (~95.7–95.9%).

In contrast, patients without these high-burden features—such as children < 10 years or those with hemoglobin ≥ 8 g/dL—showed slightly lower, though still high, concordance. These groups commonly exhibit lower peripheral blast burdens or minimal blood involvement at diagnosis; indeed, 10–20% of children with ALL lack detectable circulating blasts on initial evaluation [19], necessitating bone marrow aspiration for confirmation, particularly in resource-limited settings [4,13,20]. As expected, PB–BM concordance decreased modestly in these subgroups; however, the absolute differences remained small. For example, in the Hb ≥ 8 g/dL subgroup, the mean bias was only −0.03 (z-units), with limits of agreement of ±1.7 and overall concordance exceeding 95%. Thus, although the absence of profound anemia may indicate fewer circulating blasts, the peripheral blood still captured most leukemic populations present in the marrow.

Across the cohort, peripheral blood and bone marrow showed substantial similarity: the mean Lin’s CCC was ~0.63 (median ~0.66), with several B-lineage subpopulations exceeding 0.8. This pattern is consistent with prior large-scale studies demonstrating high diagnostic equivalence between blood and marrow in pediatric ALL. Cheng et al. reported >98% concordance in 290 paired samples [11], and Metrock et al. documented ~94% sensitivity for detecting leukemia in blood compared with marrow, with only isolated discordant cases [10]. Together, these findings support that peripheral blood generally reflects marrow immunophenotypes with high fidelity, particularly in patients with more advanced or disseminated disease.

Importantly, the global CCC (~0.63) captures variability across all 20 evaluated subpopulations and does not imply uniformly moderate agreement. Lower concordance was concentrated in maturation subsets not required for routine diagnostic classification (e.g., CD34^+^CD20^+^, CD34^−^CD19^−^, and CD34^−^HLA-DR^−^). In contrast, clinically essential markers—including CD19^+^, CD10^+^, CD34^+^, and HLA-DR^+^ consistently exceeded 95% concordance with minimal directional bias. Thus, although the global CCC reflects heterogeneity across developmental subsets, agreement in the diagnostically relevant populations remains markedly high and directly supports the practical diagnostic use of peripheral blood.

In our cohort, the clinical variables most strongly associated with high PB–BM concordance were hemoglobin < 8 g/dL, age > 10 years, and markedly elevated circulating blast burden (leukocyte or lymphocyte counts > 50,000/µL). Among the 32 evaluated patients, 47% had Hb < 8 g/dL, 44% were older than ten years, and 19% showed extreme leukocytosis or lymphocytosis; notably, 31% met at least two criteria, and 12% met all three. These proportions suggest that nearly one-third of newly diagnosed pediatric B-ALL cases may provide sufficiently representative immunophenotypes from peripheral blood alone when these indicators are present.

Although bone marrow aspirate remains the diagnostic reference, our findings outline a practical clinical framework for identifying scenarios in which peripheral blood can reliably reflect marrow immunophenotypes. PB analysis may be particularly informative when hemoglobin < 8 g/dL, age > 10 years, or marked leukocytosis/lymphocytosis (>50,000/µL) indicate extensive disease dissemination. When at least two of these conditions coexist, the probability of obtaining a representative phenotype from peripheral blood appears high, supporting its use for initial immunophenotypic assessment and reserving marrow aspiration for confirmatory or cytogenetic studies.

Beyond the analytical findings, these results have practical relevance for low- and middle-income settings such as Mexico, where limited resources and system saturation frequently delay timely treatment initiation—a factor associated with poorer survival in national cohorts [20]. Enabling earlier immunophenotypic classification through peripheral blood could help mitigate these delays and potentially improve pediatric leukemia outcomes, which still fall short of international standards [14].

## 4. Materials and Methods

### 4.1. Study Design and Setting

A comparative observational study was conducted in a pediatric population with newly diagnosed B-cell acute lymphoblastic leukemia (B-ALL) treated at the Regional Hospital of Tlalnepantla, ISSEMyM. In all cases, paired bone marrow and peripheral blood samples were obtained concurrently at diagnosis, with the objective of quantifying immunophenotypic concordance between both sources using multivariate and agreement-based methods (Bland–Altman analysis and Lin’s concordance correlation coefficient). The inclusion period spanned from January 2022 to 2024, under standardized pre-analytical and analytical conditions to ensure comparability between sample types.

### 4.2. Participants

A total of 75 pediatric patients with suspected B-ALL were evaluated, all of whom had same-day paired bone marrow and peripheral blood samples collected. Of these, 32 met all inclusion criteria and were eligible for complete immunophenotyping and DNA index assessment. The remaining cases were excluded for diagnostic or technical reasons. Some were excluded after immunophenotyping because the leukemia corresponded to T-ALL, displayed a biphenotypic or mixed-phenotype pattern, or was otherwise incompatible with B-lineage classification. Additional paired samples were discarded due to predefined laboratory quality-control criteria: insufficient pre-staining viability to obtain a reliable immunophenotype, extensive cell loss after fixation during DNA index preparation, or both. Because the study required complete same-day paired data for both analyses, any pair lacking one of the two measurements was excluded.

### 4.3. Sample Collection and Handling

Bone marrow was obtained by posterior iliac crest aspiration, and peripheral blood by venipuncture into EDTA tubes, with immediate transport to the laboratory under biosafety and traceability conditions. The pre-analytical interval from collection to staining was kept within defined, equivalent timeframes for both sample types, with recording of reception time and temperature to ensure analyte stability and comparability of measurements.

### 4.4. Flow Cytometric Immunophenotyping

Multiparametric flow cytometry was performed in an accredited clinical immunology laboratory using a Beckman Coulter CytoFLEX cytometer (3-laser, multi-color platform) (Beckman Coulter, Brea, CA, USA). Daily quality control included verification of laser performance and detector stability using CytoFLEX Daily QC Beads (Beckman Coulter, Brea, CA, USA), as recommended by the manufacturer. Compensation was assessed each day and recalibrated if deviations exceeded predefined thresholds.

For reproducibility, the antibody panel used in this study consisted of seven colors: CD10, CD19, CD20, CD22, CD34, CD45, and HLA-DR, with fluorochrome assignments exactly as listed in Supplementary Table S2. All bone marrow and peripheral blood samples were acquired under identical settings, with an acquisition threshold set on FSC and a minimum of 50,000 total events per tube. Samples were required to contain at least 500 CD45-low blast events to ensure reliable quantification; paired samples not meeting these criteria were excluded during quality review.

Data were processed using Kaluza C (Beckman Coulter, Brea, CA, USA). After debris and doublet exclusion (FSC/SSC and pulse geometry parameters), blasts were identified on CD45 vs. SSC-A, selecting CD45-low, low-complexity populations. B-lineage assignment and maturation staging were operationalized through a consistent sequential gating strategy based on the combinations CD34/CD10, CD34/HLA-DR, CD34/CD19, CD34/CD20, and CD34/CD22. Representative gates from both bone marrow and peripheral blood are shown in Supplementary Figure S1.

### 4.5. Data Management and Quality Control

Quality control included daily verification of cytometer performance and compensation using fluorescent beads, reruns when predefined deviations occurred, and exclusion of entire paired samples when blast viability, fixation performance, or panel completeness did not meet technical specifications. After applying these criteria, the analytical dataset consisted of thirty-two complete bone marrow–peripheral blood pairs, all of which contained a full and valid set of immunophenotypic markers. Consequently, no missing values were present in the final dataset, and all statistical analyses (CCC, PCA, and Bland–Altman) were performed using complete data without the need for imputation.

To confirm the stability of the results, all analyses were repeated independently on the final dataset, yielding concordant patterns in CCC estimates, PCA structure, and Bland–Altman limits of agreement. This consistency indicates that the conclusions were robust and not dependent on any single analytical run or parameter configuration.

### 4.6. Clinical Variables and Subgroups

Clinical variables extracted from medical records included age, sex, hemoglobin, leukocyte count, platelet count, lactate dehydrogenase, and organomegaly. Because clinical heterogeneity may influence the presence and representation of circulating blasts, we explored whether specific characteristics could modulate PB–BM concordance. During preliminary inspection of the dataset, several candidate variables, namely hemoglobin level, leukocyte count, platelet count, LDH, organomegaly, and different age categories, were evaluated to identify potential sources of variation.

Among these candidates, two characteristics showed the most consistent patterns: hemoglobin < 8 g/dL and age > 10 years. Both are clinically meaningful indicators of leukemic burden and dissemination, and therefore were selected for stratified analyses. All analytical procedures were repeated within each stratum using identical quality-control and preprocessing criteria. This approach was intended to assess whether PB–BM concordance remained stable across clinically distinct scenarios.

### 4.7. Statistical Analysis

All statistical analyses were performed in R (v4.3.2) within the RStudio environment (2025.09.2-418). Data preprocessing, cleaning, and integration of clinical and immunophenotypic variables were conducted with tidyverse packages (dplyr, tidyr, readr). All immunophenotypic measurements were standardized as z-scores prior to multivariate and concordance analyses to ensure comparability across markers. Principal component analysis (PCA) was performed using the FactoMineR package (2.12) and verified with the prcomp function from the stats package, examining component loadings, variance explained, and score distributions to characterize the overall structure of PB–BM similarity. No transformations beyond z-scoring were required, as alternative scaling approaches did not improve variance structure or interpretability.

Agreement between bone marrow and peripheral blood for each immunophenotypic variable was quantified using Bland–Altman analyses (blandr and BlandAltmanLeh), obtaining the mean difference (bias) and the 95% limits of agreement. Normality of PB–BM differences was evaluated via Shapiro–Wilk tests and visual inspection of Q–Q plots; distributions were sufficiently symmetric to support standard Bland–Altman limits.

Subgroup evaluations (age > 10 years and hemoglobin < 8 g/dL) were conducted using the same preprocessing steps and statistical criteria to examine whether clinical context modified PB–BM agreement.

### 4.8. Ethical Considerations

The protocol was approved by the Ethics Committee of the Regional Hospital of Tlalnepantla, ISSEMyM (approval number ISSEMYM-2020-01/BS), and was conducted in accordance with the Declaration of Helsinki. Informed consent was obtained from parents or legal guardians, and assent from minors when appropriate. Confidentiality of patient information was maintained, and data were used exclusively for research purposes.

## 5. Conclusions

Taken together, our findings show that peripheral blood can reproduce the leukemic immunophenotype observed in bone marrow with high fidelity in a significant proportion of pediatric patients with B-ALL, particularly in those with higher leukemic burden at diagnosis. This pattern reflects the biology of the disease, where extensive marrow infiltration promotes blast spillover into circulation, allowing PB to function as a phenotypic extension of the marrow compartment. However, our study is limited by its single-center design and moderate cohort size, and the analyses were restricted to the diagnostic setting; therefore, these results should not be directly extrapolated to minimal residual disease assessments or to cases with low circulating blast counts. Even so, the high levels of concordance observed, especially in clinically relevant subgroups, support the feasibility of using PB as a less invasive and more accessible alternative for initial immunophenotypic classification in pediatric B-ALL, which may be particularly valuable in health systems where timely bone marrow sampling is not always feasible.

## Data Availability

The data presented in this study are available on request from the corresponding author. The data are not publicly available due to privacy of patients.

## Supplementary Materials

The following supporting information can be downloaded at: https://www.mdpi.com/article/10.3390/ijms27010193/s1.
