# Supplementary material for: Peripheral Blood as a Diagnostic Alternative to Bone Marrow in Immunophenotyping Pediatric B-Cell Acute Lymphoblastic Leukemia
Source: Int J Mol Sci. 2025 Dec 24;27(1):193. doi: 10.3390/ijms27010193 (PMC12785395; doi:10.3390/ijms27010193)
Supplement: Supplementary file 1 [file ijms-27-00193-s001.zip › ijms-4013098-supplementary.pdf]

Supplemetary Material

Group	Mean Bias (z-score)	95% Limits of Agreement (Lower – Upper)	Overall Concordance (%)	Comment
Hemoglobin <8 g/dL	−0.012	−1.512 – 1.488	**96.8**	Highest similarity between BM and PB
Age >10 years	−0.019	−1.598 – 1.560	**96.5**	Second highest similarity
Splenomegaly	−0.024	−1.643 – 1.595	95.9	Slightly lower similarity
Hepatomegaly	−0.027	−1.687 – 1.633	95.7	Similarity slightly below splenomegaly
Age <10 years	−0.031	−1.721 – 1.659	95.4	Lower similarity
Hemoglobin ≥8 g/dL	−0.036	−1.774 – 1.702	95.2	Lowest similarity among evaluated groups

Supplementary Table S1. Concordance analysis between bone marrow and peripheral blood in different patient subgroups. The table shows the mean bias (in z-score units), the 95% limits of agreement, and the overall percentage concordance for each evaluated subgroup**.**

Antibody	Fluorochrome	Clone	Manufacturer	Catalog number	Dilution	Isotype
CD10	PB450	HI10a	BioLegend	312218	5 µl/ 1 × 10^6 cells in 100µl	IgG1 Mouse
CD19	APC	J3-119	Beckman Coulter	IM2470	5 µl/ 1 × 10^6 cells in 100µl	IgG1 Mouse
CD20	PC5	B9E9	Beckman Coulter	IM2644U	5 µl/ 1 × 10^6 cells in 100µl	IgG2 Mouse
CD22	PC7	SJ10.1H11	Beckman Coulte	B59538-AB	5 µl/ 1 × 10^6 cells in 100µl	IgG1 Mouse
CD34	PE	Imm133	Beckman Coulter	IM1420	5 µl/ 1 × 10^6 cells in 100µl	IgG1 Mouse
CD45	KrO	J33	Beckman Coulter	B59766	5 µl/ 1 × 10^6 cells in 100µl	IgG1 Mouse
HLA-DR	APC-A750	Immu-357	Beckman Coulter	B42021	5 µl/ 1 × 10^6 cells in 100µl	IgG1 Mouse

Supplementary Table S2. Antibody panels used in immunophenotyping of B-ALL patients.

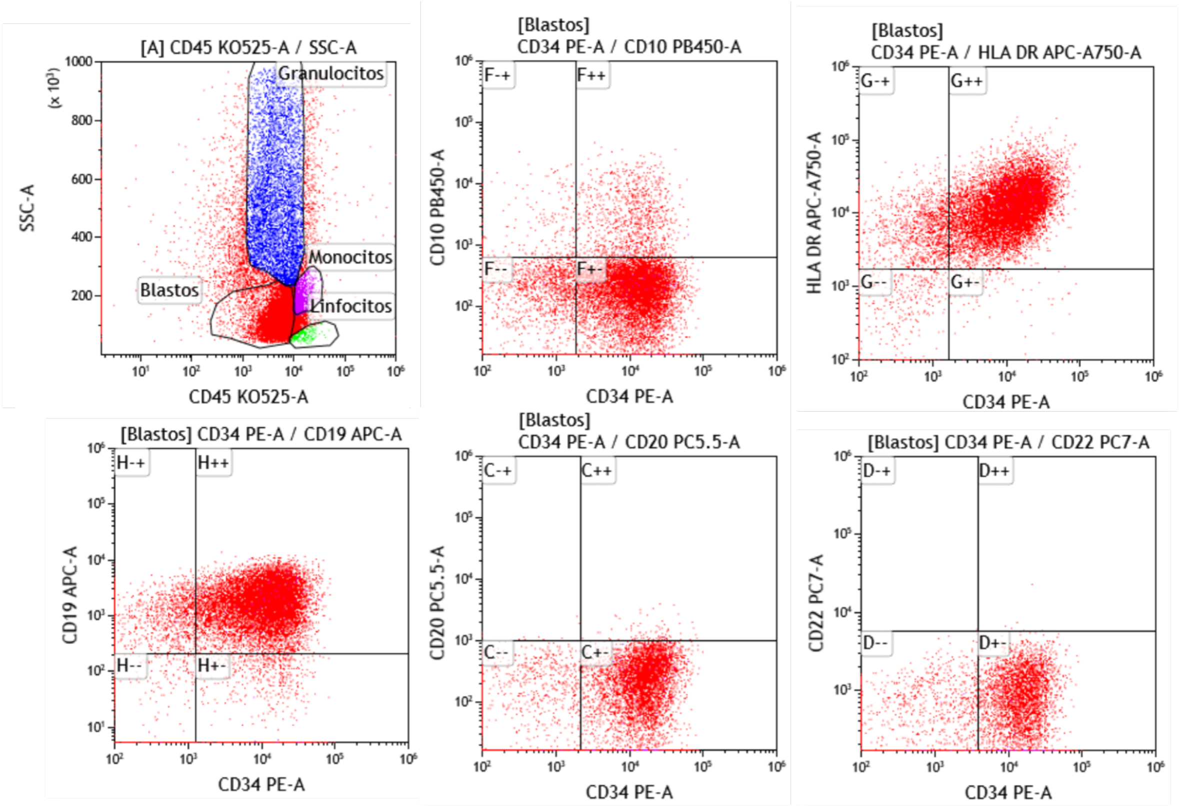

Supplementary Figure S1. Gating strategy used for immunophenotypic characterization of B-ALL blasts in paired bone marrow and peripheral blood samples. Initial identification of blast populations was performed using CD45 vs. SSC-A, followed by sequential gating based on B-lineage and maturation markers. Representative plots show CD34/ CD10, CD34/ HLA-DR, CD34/ CD19, CD34/ CD20, and CD34/ CD22 combinations, which constitute the panel used for quantitative comparison of leukemic subpopulations. All gates were applied consistently across bone marrow and peripheral blood samples. Antibody specificity and fluorochrome assignments correspond to those listed in Supplementary Table S2.
